# Pegylated liposomal doxorubicin (PLD)-containing regimen as a novel treatment of monomorphic epithelial intestinal T-cell lymphoma (MEITL): A case report and review of literature

**DOI:** 10.1097/MD.0000000000031326

**Published:** 2022-11-04

**Authors:** Yue Chen, Hongzhi Xu, Ningning Shan, Huiting Qu

**Affiliations:** a Department of Hematology, Shandong Provincial Hospital Affiliated to Shandong First Medical University, Jinan, Shandong, China; b Department of Hematology, Shandong Provincial Hospital, Shandong University, Jinan, Shandong, China.

**Keywords:** MEITL, Diagnosis, Treatment, Pegylated Liposomal Doxorubicin

## Abstract

Monomorphic intestinal T-cell lymphoma (MEITL) is a rare, aggressive peripheral T-cell lymphoma that arises from intestinal epithelial lymphocytes. Currently, MEITL lacks standard treatment options. Under the current treatment regimen, the median survival time for patients is only 7 months. Chemotherapy followed by hematopoietic stem cell transplantation may improve patient outcomes. New anti-lymphoma drugs, including chidamide and PEG-asparaginase, are being tested against MEITL. To our knowledge, there are currently no data on the pegylated liposomal doxorubicin (PLD) regimen for MEITL therapy.

Patient concerns, diagnosis and interventions: We report the case of a 54-year-old patient diagnosed with MEITL who presented with abdominal pain and was treated with a cyclophosphamide, doxorubicin, vincristine, prednisone, etoposide regimen containing PLD.

**Outcomes:**

After 15 months of follow-up, the patient is currently alive and disease free. The application of doxorubicin liposomes in chemotherapy regimens may be a new way to treat MEITL.

**Review:**

We searched the literature on MEITL and selected 52 case reports. We summarized the clinical characteristics and treatment of 53 patients (including the current patient).

**Conclusion:**

It highlights 2 important clinical findings. First, for patients with MEITL treated with the cyclophosphamide, doxorubicin, vincristine, prednisone, etoposide regimen, PLD has fewer adverse reactions and better long-term survival than doxorubicin. Second, an early diagnosis is necessary for prompt treatment. We believe that this manuscript will be valuable to all the researchers who are interested in.

## 1. Introduction

Monomorphic intestinal T-cell lymphoma (MEITL) is a malignant tumor of extra nodal lymphoid tissue caused by proliferation of intraepithelial lymphocytes. MEITL is a relatively rare disease previously known as type II of Enteropathy associated T-cell lymphoma, accounting for approximately 5% of gastrointestinal lymphomas and less than 1% of all non-Hodgkin lymphomas.^[1]^ MEITL has been independently identified as a subtype of lymphoma by the World Health Organization (WHO) since 2017. MEITL currently lacks a standard treatment regimen, and usually follows the cyclophosphamide, doxorubicin, vincristine, prednisone, etoposide (CHOPE) regimen for T-cell lymphoma. However, the curative effect of current chemotherapy is not good. After chemotherapy, the median survival time is only 7 months. Hematopoietic stem cell transplantation may improve outcomes in patients with MEITL. In addition, new drugs including Chidamide and PEG-Asparaginase are in clinical trials for MEITL. Here, we introduce a case of MEITL patients treated with CHOPE regimen using doxorubicin liposome instead of doxorubicin.

## 2. Case report

A previously healthy 54-year-old male patient, presented with the upper abdominal pain for 1 month and the weight loss of about 10 kg for 6 months. He denied night sweat, fever and denied any tobacco or alcohol use. His family history was not notable. Initial vital signs at the physical examination were as follows: blood pressure, 105/65 mm Hg; heart rate, 76 beats/minute; respiratory 19 times/minute. The abdomen was soft, no obvious mass was touched on the abdomen, no swollen lymph nodes were touched, mild tenderness in the upper abdomen, no rebound pain.

**Figure 1.**
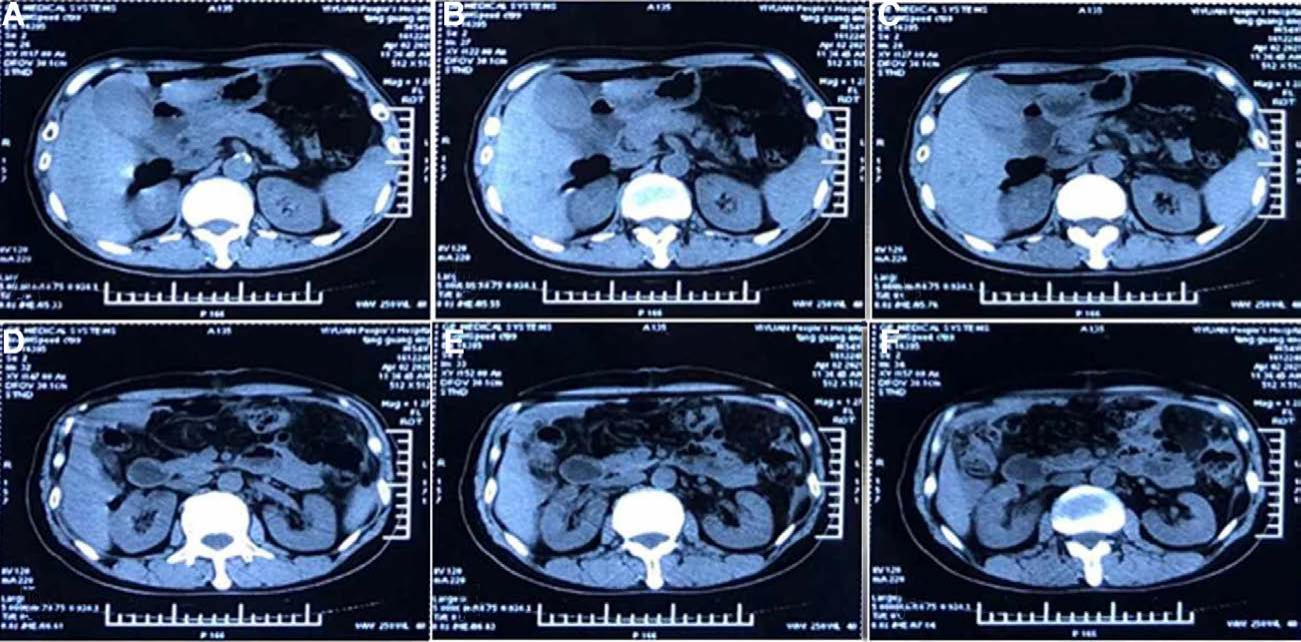
(A–F): Abdominal CT with enhancement: uneven thickening of intestinal wall, consistent with malignant tumor. CT = computed tomography.

**Figure 2.**
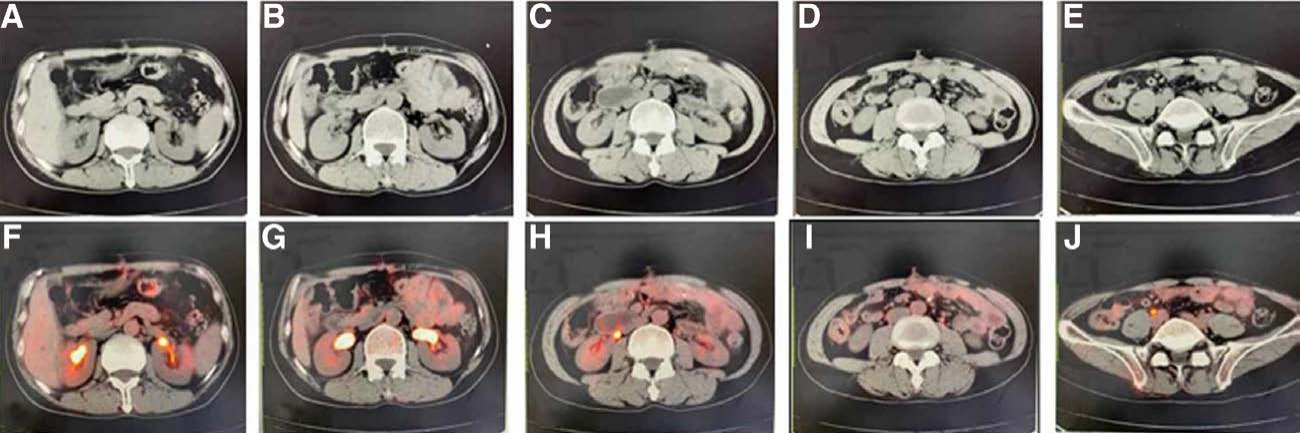
(A–J): PET-CT: no abnormal radioactive distribution was found at the corresponding place. Cord shadow can be seen under the skin of the abdominal wall operation area, with slightly high radiation distribution, and the highest SUV is 1.5. PET-CT = positron emission computed tomography.

Laboratory Examination showed no obvious abnormalities. X-ray of the thorax and lymph node sonography were unremarkable. Abdominal computed tomography (CT) with enhancement showed uneven thickening with the small bowel (Fig. 1A–F). The patient underwent an open small intestinal partial, transverse partial resection. These lesions were biopsied. An extensive lymphocytic infiltrate of atypical was observed in the biopsies from the small intestine and transverse colon with invasive growth and infiltration into the whole intestinal layer. The intestines adjacent to the tumor were without lymphocytic infiltrate abnormalities and so did the peripheral lymph nodes. Histopathologic examination of the tumor revealed sheets of relatively monotonous medium-sized cells with round or irregular nuclei, intensely concentrated chromatin and rim of pale cytoplasm. The tumor cells were positive for CD20, CD3 and CD43. But it is negative for CD5, CD10, PAX-5, MUM1, and BCL6. Ki-67 demonstrated a high proliferation index of 40% to 50%. In situ hybridization for Epstein–Barr virus remained negative. Positron emission computed tomography showed postoperative intestinal lymphoma, and no abnormal radioactive distribution was found at the anastomosis of intestinal lymphoma operation. Cord shadow can be seen under the skin of the abdominal wall operation area, with slightly high radiation distribution, and the highest SUV is 1.5 (Fig. 2A–J). The clinical findings, morphologic appearance as well as the immunohistochemistry staining pattern were consistent with the diagnosis of MEITL. Bone marrow aspiration and biopsy did not show any evidence of lymphoma. The patient was diagnosed as MEITL in Ann Arbor stage IV.

The patient with MEITL were treated with the pegylated liposomal doxorubicin (PLD) containing CHOPE regimen consisting of an intravenous administration of Cyclophosphamide (750 mg/m^2^), Liposomal doxorubicin (35 mg/m^2^), and Vincristine (1.4 mg/m^2^) on d1, an oral administration of Prednisolone (60 mg/m^2^) on d1–5, as well as an intravenous administration of Etoposide (50 mg/m^2^) on d1–3. The treatment with PLD 35 mg/m^2^ was initiated in April 2021 and performed every 3 weeks, and a total of 5 cycles was performed. Interim assessment was done after 3 cycles of CHOPE regimen. His bellyache subsided significantly after completion at interim assessment. Abdominal CT scan after 3 cycles of CHOPE showed no evidence of lymphoma. No enlarged lymph nodes on physical examination and ultrasonic instrument were observed. He achieved complete remission (CR) after 3 full cycles of CHOPE therapy containing PLD. Because of these impressive results, the patient continued the 2-cycle CHOPE regimen consolidation therapy. Monitored by regular laboratory investigations and electrocardiography, adverse reactions were not observed after chemotherapy drugs including PLD therapy. There were no serious chemotherapy-related adverse events, except nausea and vomiting. After 5 cycles of CHOPE regimen, which were well tolerated without grade 3 or 4 toxicities, no further deterioration of disease had been measured. And this is different from the regimen containing doxorubicin. In December 2021, the patient was followed up regularly after discharge, and no lymphoma progression was found. The median overall survival of patients receiving anthracycline-containing chemotherapy was 7 months.^[2]^ The last follow-up was accomplished by telephone in May 2022. The patient has been in CR for 15 months after the initial diagnosis.

## 3. Literature review

We performed a literature review of previous MEITL cases using the search terms “MEITL”, “type II of enteropathy associated T-cell lymphoma” and “intestinal lymphoma”. We performed a retrospective review of MEITL from January 2016 to June 2021 and identified 52 cases of MEITL with relatively detailed medical history reported in the English literature to date. The clinical characteristics and treatment of 53 patients (including the current patient) are summarized in Table 1.^[3-32]^ Patients ranged in age from 35 to 88 years, and 71.7% were male. Among the reported cases, the main symptoms were abdominal pain (43.4%), diarrhea (41.5%) and weight loss (35.8%). In addition, 1 patient had small bowel obstruction and/or gastrointestinal bleeding, and 2 MEITL patients had bowel perforation. Interestingly, 1 patient who was initially admitted to the hospital for a urinary tract infection turned out to be MEITL. Of these cases, the jejunum was involved in 11 cases, the duodenum was involved in 8 cases, the ileum was involved in 5 cases, and the remaining 26 cases included the unspecified small bowel site in this case. In addition, 5 cases had evidence of bone marrow involvement, 4 cases reported evidence of pulmonary infiltrates and 1 had evidence of brain involvement. Treatment options mainly include various combination chemotherapy regimens. Approximately 30.2% of reported patients received cyclophosphamide, vincristine, doxorubicin, and prednisone (CHOP) and 15.1% received etoposide, prednisone, vincristine, cyclophosphamide, and doxorubicin and/or CHOPE. Only our case was treated with PLD instead of doxorubicin with a CHOPE regimen. Unfortunately, despite prompt treatment, the prognosis remains poor, and outcomes are poor, with a mortality rate of 51.1% within the first year after diagnosis.

**Table 1:** Literature review of previous reported cases of monomorphic epitheliotropic intestinal T-cell lymphoma (MEITL). (2016–2021).

Authors (yr)	Age and gender	Clinical presentation	Clinical stage of MEITL	Intestinal localization	Other involved sites	Chemotherapy	Follow-up
Chen et al^[11]^ (2016)	60 yr/o, male	Abdominal pain	I A	Small bowel	–	CHOP + IVE/MT × 3 cycles + GDP	Died after 2 and a half years
Ishibashi et al^[12]^ (2016)	60 yr/o, male	Persistent diarrhea and weight loss	IV B	Duodenum, jejunum and cecum	Stomach bone marrow	CHASE	Died after 3 years
Ishibashi et al^[12]^ (2016)	40 yr/o, female	Diarrhea and weight loss	I B	Small bowel and colon	–	THP-COP	Died after 1 year
Ishibashi et al^[12]^ (2016)	50 yr/o, female	Abdominal distention	IV A	Small bowel, colon and rectum	Bone marrow	CHOP + high dose MTX/cytarabine	Died after 9 mos.
Ishibashi et al^[12]^ (2016)	70 yr/o, male	Nausea	IV A	Small bowel, colon, cecum and rectum	Bone marrow	SMILE	Died after 9 mos.
Hong et al^[10]^ (2016)	67 yr/o, male	Abdominal pain, night sweating and weight loss	II B	Terminal ileum, cecum and colon	–	4 cycles of CHOP	Died after 4 mos.
Hong et al^[10]^ (2016)	50 yr/o, male	Abdominal pain and low grade fever	II A	Ileum and colon	-	6 cycles of R-CHOP	Alive, 60 mos.
Hong et al^[10]^ (2016)	48 yr/o, male	chronic diarrhea and weight loss	Not given	Jejunum	-	Could not undergo chemotherapy	Died after 14 mos.
Hong et al^[10]^ (2016)	55 yr/o, female	Chronic diarrhea, abdominal pain, and weight loss	III B	Duodenum and jejunum	-	CHOP and chemotherapy regimen was changed (regimen not specified)	Died after 13 mos.
Liong et al^[13]^ (2016)	50 yr/o, male	Chronic diarrhea, and weight loss	IV B	Small bowel	Lung, spleen and bone marrow	2 cycles of cyclophosphamide, hydroxydaunorubicin, oncovin and prednisolone and a cycle of ICE	Died after 4 mos.
Nozari et al^[14]^ (2017)	35 yr/o, male	Chronic diarrhea	I A	The small intestine	–	1 cycle of CHOP	Died after 1 mo
Komeda et al^[15]^(2017)	65 yr/o, male	Left-sided ulcerative colitis	II	The small and large intestines	–	5 course of chemotherapy (regimen not specified)	Died after 7 mos.
Aiempanakit et al^[16]^(2017)	67 yr/o, male	Chronic diarrhea and weight loss	IV B	Duodenum and jejunum	The abdominal and chest walls	Anthracycline-based chemotherapy	Died after 2 mos.
Chan et al^[17]^(2017)	44 yr/o, male	Abdominal pain	I A	Small bowel	–	SMILE	Died after 136 mos.
Chan et al^[17]^(2017)	50 yr/o, male	Abdominal distension	II A	Small bowel	–	SMILE	Died after 3 weeks
Chan et al^[17]^(2017)	55 yr/o, male	Ascites and intestinal obstruction	IV A	Small bowel	Peritoneum, lung nodules and SCF LN	Dexamethason, etoposide, ifosfamide, L-asparaginase, cisplatin and gemcitabin	Died after 1 month
Chan et al^[17]^(2017)	57 yr/o, female	Abdominal pain	II A	Small bowel	-	Dexamethason, etoposide, ifosfamide, L-asparaginase, cisplatin and gemcitabin	Died after 11 mos.
Chan et al^[17]^(2017)	39 yr/o, male	Diarrhea	IV A	Small bowel and large bowel	Peritoneum, PT LN, IM LN, SCF LN, diaphragm and pleura	CHOP	Died after 3 mos.
Chan et al^[17]^(2017)	61 yr/o, female	Abdominal pain and diarrhea	I A	Large bowel	–	SMILE	Died after 16 mos.
Chan et al^[17]^(2017)	51 yr/o, female	Intestinal perforation	III A	Small bowel	–	Dexamethason, etoposide, ifosfamide, L-asparaginase, cisplatin and gemcitabin	Died after 20 mos.
Chan et al^[17]^(2017)	70 yr/o, female	Abdominal pain	I A	Duodenum	–	Dexamethason, etoposide, ifosfamide, L-asparaginase, cisplatin and gemcitabin	Died after 6 mos.
Chan et al^[17]^(2017)	51 yr/o, male	Diarrhea and wight loss	II B	Small bowel	–	CHOP	Died after 14 mos.
Chan et al^[17]^(2017)	65 yr/o, male	Gastrointestinal bleeding	IV A	–	Stomach, esophagus and lung	Dexamethason, etoposide, ifosfamide, L-asparaginase, cisplatin and gemcitabin	Died after 13 mos.
Chan et al^[17]^(2017)	57 yr/o, male	Abdominal pain	II A	Small bowel	–	SMILE	Alive, 13 mos.
Chan et al^[17]^(2017)	68 yr/o, male	Gastrointestinal pain	II A	Large bowel	–	Dexamethason, etoposide, ifosfamide, L-asparaginase, cisplatin and gemcitabin	Alive, 13 mos.
Antoniadou et al^[18]^(2017)	76 yr/o, male	Severe dyspnea	IV A	Jejunum	Pleura	–	Died after 1 month
Gentille et al^[8]^(2017)	70 yr/o, female	Abdominal pain, nausea, vomiting, diarrhea, and weight loss	Not given	Jejunoileal junction and colon	–	4 cycles of EPOCH	Died after 17 mos.
Zhao et al^[19]^(2018)	47 yr/o, female	Diarrhea and weight loss	II B	Rectum, sigmoid colon, and distal ileum	–	Could not undergo chemotherapy	Died after 4 mos.
Aoyama et al^[20]^(2018)	83 yr/o, male	Fever and diarrhea	II B	Colon	–	CHOP followed by DeVIC	Died: no stated time frame
Tian et al^[6]^(2019)	58 yr/o, male	Abdominal pain, diarrhea and weight loss	IIB	Colon and rectum	stomach	1 cycles of CHOP	Died after 2 mos.
Tian et al^[6]^(2019)	64 yr/o, female	Chronic diarrhea and weight loss	II B	Small bowel and colon	–	Romidepsin with revlimid	Died after 7 mos.
Ikeda et al^[21]^(2019)	61 yr/o, male	3 episodes of ileal strangulation	IV	Ileum	Bone marrow and pleura	2 cycles of CHOP and 1 cycle of ICE	Died after 3 mos.
Kubota et al^[22]^(2019)	41 yr/o, male	Diarrhea and abdominal pain	IV A	An extramural tumor of the small intestine	Cerebrospinal flfluid	CHOP and 3 cycles of ICE chemotherapy, intrathecal chemotherapy and high-dose chemotherapy	Complete remission (CR)
Noh et al^[23]^(2019)	88 yr/o, male	Nausea, vomiting, and weight loss	Not given	Duodenum	–	Chemotherapy (regimen not specified)	Not given
Liu et al^[24]^(2020)	43 yr/o, female	Abdominal pain and weight loss	I B	duodenum	–	4 cycles of the CHOPE and 2 cycles of the DHAP with chidamide	11 mos. since diagnosis
Fei et al^[25]^(2020)	58 yr/o, male	Persistent abdominal pain and weight loss for 3 mos.	Not given	Small intestine	–	3 cycles of EPOCH cycles	7 mos. since diagnosis
Fei et al^[25]^(2020)	57 yr/o, male	Diarrhea and weight loss for 3 mos.	Not given	Small intestine, cecum, colon and rectum	–	6 cycles of CHOP	8 mos. since diagnosis
Fei et al^[25]^(2020)	80 yr/o, male	Small intestine perforation	Not given	Small intestine	-	Not given	Not given
Fei et al^[25]^(2020)	67 yr/o, male	Abdominal mass	Not given	Duodenum and jejunum	–	Not given	Not given
Fei et al^[25]^(2020)	61 yr/o, male	Upper abdominal pain and intermittent black stool	Not given	Small intestine	–	2 cycles of CHOP combined with chidamide, CHOPE combined with chidamide for 4 cycles	15 mos. since diagnosis
Liu et al^[9]^ (2020)	35 yr/o, female	Abdominal distention, abdominal pain and high fever	Not given	Small intestine and colon	–	IVE and chidamide for 6 cycles	17 mos. since her diagnosis
Gopalakrishna et al^[3]^(2020)	53 yr/o, male	Abdominal pain, fever, burning sensation and increased urinary	II B	Jejunum	–	3 cycles of CHOPE	Not given
Suzuki et al^[26]^(2020)	74 yr/o, male	Diarrhea	IV A	Small intestine	The right cerebral hemisphere, bilateral lungs and pelvic cavity	Could not undergo chemotherapy	Not given
Susu Lu et al^[27]^(2020)	65 yr/o, male	Chronic diarrhea edema	I A	Colon	Stomach	3 cycles of CHOP	Died after 13 mos.
Chuah et al^[28]^(2020)	36 yr/o, male	Intractable diarrhea and weight loss	I B	The duodenojejunal junction	–	7 cycles of CEOP, 2 cycles of MINE and ESHAP	Died after 9 mos.
Afzal et al^[4]^(2020)	39 yr/o, male	Abdominal pain and intermittent diarrhea	IV A	Jejunum	The bilateral adrenal glands	Chemotherapy (regimen not specified)	Died after 7 mos.
Mago et al^[29]^(2021)	59 yr/o, male	Intermittent shortness of breath exacerbated, abdominal distention and night sweats	IV B	Duodenum and jejunum	Pleura	CHOPE	Few days since diagnosis
Morimoto et al^[5]^(2021)	74 yr/o, female	Abdominal distension and melena	IV A	Small intestine	Brain	1 cycle of CHOP and 2 cycles of ESHAP	Died: no stated time frame
Ozaka et al^[7]^(2021)	68 yr/o, female	Melena	I A	Duodenum	–	8 full cycles of CHOP	Alive, 8 mos.
Zhong et al^[30]^(2021)	49 yr/o, male	Abdominal pain, diarrhea, fatigue and fever	IV B	The ileocecal region and colon	Stomach, appendix, liver and spleen	-	2.5 mos. since diagnosis
Aoki et al^[31]^(2021)	77 yr/o, female	Abdominal pain, night sweats and fever	IV B	Jejunum and ileum	The ribs, spine and pelvic bone	EPOCH	Alive, 1 year
Kansoun et al^[32]^(2021)	59 yr/o, male	Weight loss, recurrent nocturnal episodes of fever, night sweats and abdominal pain	I B	Small bowel and the sigmoid	Appendix and urinary bladder	6 courses of adjuvant chemotherapy according to the following regimen: brentuximab vedotin in combination with cyclophosphamide, doxorubicin, and prednisone.	Alive, beyond 1 year
Current patient	54 yr/o, male	The upper abdominal pain and weight loss	IV B	Small intestinal and colon	–	5 cycles of CHOPE (liposomal doxorubicin)	Alive, 13 mos.

CEOP = cyclophosphamide, etoposide, vincristine and prednisone, CHASE = cyclophosphamide, cytarabine, etoposide, and dexamethasone, CHOP = cyclophosphamide, doxorubicin, vincristine, and prednisone, CHOPE = CHOP and etoposide, DeVIC = etoposide, doxorubicin, oncovin, and prednisolone, EPOCH = etoposide, prednisone, vincristine, cyclophosphamide, and doxorubicin, ESHAP = ifosfamide, cisplatin, etoposide, methylprednisolone, and cytarabine, GDP = gemcitabine, dexamethasone, and cisplatin, ICE = ifosfamide, carboplatin and etoposide, IM = internal mammary, IVE = ifosfamide, vincristine, and etoposide, LN = lymph node, MEITL = monomorphic epitheliotropic intestinal T-cell lymphoma, MINE = mesna, ifosfamide, mitoxantrone, etoposide, mos. = months, MTX = methotrexate, PT = paratracheal, R-CHOP = rituximab, cyclophosphamide, doxorubicin, vincristine, and prednisone, SCF = supraclavicular fossa, SMILE = dexamethasone, methotrexate, ifosfamide, L-asparaginase, and etoposide, THP-COP = pirarubicin, cyclophosphamide, vincristine, and prednisolone, y/o = years old.

## 4. Discussion

MEITL is a rare primary intestinal T-cell lymphoma, known as an aggressive T-cell lymphoma with a poor prognosis and high mortality rate. In the 2008 edition of the WHO classification of neoplasms, enteropathy-associated T cell lymphoma was classified as classical (Type I) and monophasic (Type II).^[33]^ Type I cells have significant polymorphism. Type II cells have medium size and relatively single morphology and more common in the Asian population.^[3,34]^ Although the 2 diseases share many common clinical and pathological features, they have their own characteristics in terms of epidemiology, histology, immunophenotype and genetic characteristics. The WHO classification scheme of 2017 recognizes 4 subtypes of intestinal T-cell lymphoma: enteropathy-associated T-cell lymphoma, monomorphic epitheliotropic intestinal T-cell lymphoma, intestinal T-cell lymphoma, not otherwise specified, and indolent T-cell lymphoproliferative disorder of the GI tract (provisional).^[4]^

MEITL occurs at a wide range of ages with a median age of 58 years and with a male to female ratio of 2:1.^[35]^ MEITL is an aggressive lymphoma that involves not only the gastrointestinal tract, but also extensive systemic involvement, and there are case reports of brain involvement.^[5]^ It is generally believed that patients with MEITL do not have celiac disease, but there are also reports in the literature that celiac disease is not a specific sign to identify MEITL.^[36]^ Patients with MEITL lack specific endoscopic findings, so diagnosis relies on pathological diagnosis and immunohistochemistry.^[6]^ The prognosis of MEITL is poor with a previously reported median overall survival of only 7 months^[37]^ and 1-year overall survival is only 36%.^[35]^

Doxorubicin, an anthracycline antitumor agent, is widely used in the treatment of malignant tumors such as lymphoma. The CHOP regimen (cyclophosphamide, vincristine, doxorubicin, prednisone) is a widely used treatment regimen for T-cell lymphoma. Unfortunately, the long-term use of this drug is limited by cardiac toxicity, especially drug-induced congestive heart failure, even chronic irreversible cardiotoxicity. Due to the cardiotoxicity of cumulative dose limitation, doxorubicin has been formulated in liposomes to selectively deliver the drug to tumors and limit the accumulation of drug in healthy tissues, especially in the heart. Wrapping doxorubicin in the liposome will significantly change its bioavailability and biological distribution, so as to change its biological activity. PLD has been shown to significantly reduce cardiotoxicity and is effective against tumors. To our knowledge, there is currently no standard treatment regimen for MEITL, and the CHOPE regimen containing doxorubicin is less effective. Here, we report a patient with pathologically confirmed MEITL treated with a CHOPE regimen containing liposome doxorubicin.

There are no specific guidelines for the treatment of MEITL. There is a report in the literature that a patient survived for 5 years after early diagnosis and 8 cycles of CHOP regimen.^[7]^ Various new treatments have been tried in MEITL, including chidamide and PEG-asparaginase,^[8]^ but chidamide does not appear to improve patient survival.^[9]^ Current consensus suggest a systemic anthracycline-containing chemotherapy with primary surgical resection to be an effective regimen.^[8]^ A multicenter retrospective study found that the CHOP regimen in MEITL was insufficient to achieve CR, which may be an important factor affecting survival.^[38]^ Hematopoietic stem cell transplantation may bring survival benefits to patients with MEITL, but there are also reported cases of early central nervous system (CNS) relapse after cord blood transplantation in the literature.^[39]^

We report a case of MEITL diagnosed with CHOPE regimen containing PLD. Our summary in this case emphasizes 2 important clinical points. Firstly, for patients with MEITL treated with CHOPE regimen, PLD has less adverse reactions and long-term survival than doxorubicin. Secondly, there are few relevant reports and lack of characteristic clinical manifestations features, so the diagnosis is very challenging. It is easy to misdiagnosis and mistreatment, and the disease is invasive and progresses rapidly, with high mortality and very poor clinical prognosis.^[4]^ Effective treatment administered early is critical in order to long-term survival.

Doxorubicin is an active chemotherapeutic agent for MEITL, which shows efficacy in different types of cancer. But it can cause some adverse effects, such as myelosuppression, as well as heart and digestive system toxicities, dose-dependent cardiac toxicity is a main side effect of doxorubicin, especially in elderly patients where there is a high rate of preexisting cardiac disease.^[40]^ Based this, Duncan found liposomal formulations with slow release of doxorubicin were developed and successfully reduced the cardiac toxicity in cancer treatment (Duncan, 2006; Gabizon and Papahadjopoulos, 1988; Gill et al, 1995). In our case report, after surgical resection, PLD containing CHOPE regimen was given. PLD has a stable spatial structure and encapsulated form. Its binding to plasma proteins is reduced. Compared to conventional doxorubicin injection, PLD has longer plasma circulation, lower clearance rate, higher blood concentration, and longer t1/2 (68 hours).^[41]^ PLD has a longer retention time in blood circulation and releases more slowly, which can act directly on tumor tissues. Therefore, it can reduce the toxicities associated with doxorubicin, especially the myelosuppression and cardiotoxicity, and make doxorubicin more targeted delivery. Compared with doxorubicin solution, PLD have produced significant efficacy and toxicity profiles in solid tumor and lymphoma patient. The patient has survived for a year and has no adverse events of cardiotoxicity after chemotherapy drugs.

Although MEITL is an aggressive T-cell lymphoma with progresses rapidly, an extremely poor prognosis and high mortality rate, patients with MEITL might survive for a long term if effective treatment is administered early. In our case, the patient presented with nonspecific clinical manifestations, yet total excision revealed early stage of MEITL. The operation and diagnosis were timely and the treatment was positive. Therefore, it is significant to diagnose and treat at an early stage. Embarrassed the diagnosis of MEITL, it is difficult and prone to missed diagnosis, misdiagnosis and mistreatment. The main reasons for the difficult diagnosis of MEITL are as follows: A. The low incidence of MEITL had not attracted the attention of medical researchers and practitioners. B. The clinical manifestations of MEITL are nonspecific and mainly digestive tract symptoms. It is difficult to distinguish from other diseases. Diarrhea is the first symptom, and there will be symptoms such as abdominal pain, abdominal distension, abdominal mass, and changes in defecation rules. Other nonspecific clinical manifestations: hypoalbuminemia, anemia, etc. Systemic symptoms can appear fever, fatigue, poor appetite, progressive emaciation, etc. C. Small intestinal CT is helpful to detect lesions, and it is also helpful to judge the scope of the affected intestine, providing guidance for the next endoscopic examination. However, small intestinal CT lacks specificity and has a certain false-negative rate for MEITL diagnosis. No abnormality found under CT cannot completely exclude MEITL, so it plays a sufficient and unnecessary role in the diagnosis of MEITL. For endoscopic manifestations, MEITL may have mild and varied early mucosal changes, such as superficial ulceration, peripherally or 1/2 circumferential lumen, granular changes, and Mosaic signs.^[10]^ D. In the majority of MEITL, the lymphoma cells are positive for CD2, CD3, CD7, CD8 and CD56, and negative for CD4, CD5 and CD30.^[2,42,43]^ However, immunophenotypic variation occurs; some cases are negative for CD8 and/or CD56, and a minority co- expresses CD4 and CD8.^[2,42,43]^ The diagnosis of MEITL needs to comprehensively and carefully perform auxiliary examinations and clinical manifestations to rule out MEITL. Abnormally high uptake of fluorodeoxyglucose (I8F-FDG) in tumor tissue during positron emission computed tomography may be helpful in diagnosing lymphoma.

Treatment with PLD containing CHOPE is very rare in MEITL. Using the CHOPE regimen containing PLD is our new attempt in MEITL therapy. Would PLD offers an advantage for survival? It remains questionable. It is needed that further investigate and evaluate toxicity and efficacy of liposomal doxorubicin in the treatment of MEITL for an optimal treatment algorithm. Further investigations with more patients and randomized controlled studies in comparison to the Doxorubicin are warranted. Given that MEITL is a rare and newly proposed disease, international and multi-center efforts are needed to conduct prospective studies.

## Acknowledgements

This work was supported by grants from Natural Science Foundation of Shandong Province (ZR2021MH319).

## Author contributions

**Conceptualization:** Hongzhi Xu, Ningning Shan, Huiting Qu.

**Data curation:** Yue Chen, Ningning Shan, Huiting Qu.

**Investigation:** Yue Chen, Hongzhi Xu, Huiting Qu.

**Project administration:** Yue Chen, Huiting Qu.

**Supervision:** Hongzhi Xu, Huiting Qu.

**Writing – review & editing:** Yue Chen, Hongzhi Xu, Ningning Shan, Huiting Qu.
